# Measuring the Interactions Between Health Demand, Informatics Supply, and Technological Applications in Digital Medical Innovation for China: Content Mapping and Analysis

**DOI:** 10.2196/26393

**Published:** 2021-07-06

**Authors:** Jian Du, Ting Chen, Luxia Zhang

**Affiliations:** 1 National Institute of Health Data Science Peking University Beijing China; 2 Institutes of Science and Development Chinese Academy of Sciences Beijing China

**Keywords:** medical informatics, Medical Subject Headings (MeSH), health demand, informatics supply, technological applications

## Abstract

**Background:**

There were 2 major incentives introduced by the Chinese government to promote medical informatics in 2009 and 2016. As new drugs are the major source of medical innovation, informatics-related concepts and techniques are a major source of digital medical innovation. However, it is unclear whether the research efforts of medical informatics in China have met the health needs, such as disease management and population health.

**Objective:**

We proposed an approach to mapping the interplay between different knowledge entities by using the tree structure of Medical Subject Headings (MeSH) to gain insights into the interactions between informatics supply, health demand, and technological applications in digital medical innovation in China.

**Methods:**

All terms under the MeSH tree parent node “Diseases [C]” or node “Health [N01.400]” or “Public Health [N06.850]” were labelled as H. All terms under the node “Information Science [L]” were labelled as I, and all terms under node “Analytical, Diagnostic and Therapeutic Techniques, and Equipment [E]” were labelled as T. The H-I-T interactions can be measured by using their co-occurrences in a given publication.

**Results:**

The H-I-T interactions in China are showing significant growth and a more concentrated interplay were observed. Computing methodologies, informatics, and communications media (such as social media and the internet) constitute the majority of I-related concepts and techniques used for resolving the health promotion and diseases management problems in China. Generally there is a positive correlation between the burden and informatics research efforts for diseases in China. We think it is not contradictory that informatics research should be focused on the greatest burden of diseases or where it can have the most impact. Artificial intelligence is a competing field of medical informatics research in China, with a notable focus on diagnostic deep learning algorithms for medical imaging.

**Conclusions:**

It is suggested that technological transfers, namely the functionality to be realized by medical/health informatics (eg, diagnosis, therapeutics, surgical procedures, laboratory testing techniques, and equipment and supplies) should be strengthened. Research on natural language processing and electronic health records should also be strengthened to improve the real-world applications of health information technologies and big data in the future.

## Introduction

### Background

Medical informatics (MI), or biomedical and health informatics, has become an established scientific discipline worldwide. It studies the data, information, and knowledge of biomedicine and health care and their systematic organization, representation, and analysis methods [1]. Basic research scholars in this community adopt quantitative and qualitative methods for understanding and improving the process surrounding the use of information, with the specific goal of advancing biomedical science, whereas applied research scholars leverage information technologies to improve health care outcomes [2]. The application of health information technology (HIT) was proposed as a promising potential solution for improving the productivity, effectiveness, and quality of health care services. The most important benefits of HITs are to reduce medical errors and costs, improve patients’ quality of life, and enhance medical decision making. Informatics with big data can be exploited for a wide variety of applications including artificial intelligence (AI), predictive analytics, and point-of-care clinical decision making [3]. The United States has twice promoted HIT through legislation, including the Health Insurance Portability and Accountability Act (HIPAA) in 1996 and Health Information Technology for Economic and Clinical Health (HITECH) in 2009 [4].

In China, there are also 2 major government incentives in the development of MI. One is the launch of the second round of medical reform in 2009 when a substantial investment was put into MI [5]. An important contribution of this health care reform is that MI has been defined as one of the “four constructs and eight pillars,” which is the foundation of this reform. As a result of these health policies, the Chinese government and industry have invested heavily in hospital informatics and population health informatics. The second incentive is that in 2016, China released its first health initiative, Healthy China 2030, which guides and coordinates a nationwide strategy for improving China’s population health and the national health system. China aims to establish a comprehensive health information system in all public hospitals and primary health care facilities and to develop “Internet + Health initiatives” by using new internet-based technologies to increase access to health care and improve the quality and efficiency of health care delivery. In particular, telemedicine was encouraged as a means toward connecting residents with public hospitals, and its use was viewed as a way to reduce inequity between urban and rural areas apart from improving access to health care. Starting with the experience of fighting COVID-19, China is speeding up its efforts in the use of cutting-edge information technologies in medicine and health care, with the aim of innovating the management and service mode, optimizing the allocated resources, and improving service efficiency [6].

However, evidence shows that there is an imbalance between research and practice of MI in China. This discipline had long been focused on library-oriented informatics instead of hospital-oriented informatics. Academic MI research lags behind HIT applications in China. Current MI in China can be described as “hot in industrial HIT applications and cold in academic research.” This increased focus on HIT applications rather than MI research has hampered the applications of theoretical research to a real-world setting, resulting in repeated HIT construction and huge resource waste in China [7-9].

To characterize the landscape of academic research in MI around the world or in China, previous researchers either used publication data collected in terms of informatics-related concepts, such as the Medical Subject Headings (MeSH) “Public Medical informatics” [10,11], or specialty journals [12]. However, MI is a multidisciplinary field; data from specialty concepts and journals may not reflect the activities outside of the MI communities. Thus, it is difficult to depict a complete picture about the pattern of interactions between informatics concepts or techniques and health or medical needs. To the best of our knowledge, there have been limited systematic investigations of the interactions between health demand and informatics supply in China.

To fill this gap, this paper proposes a new approach to collecting research publications with a broad interpretation of the MI using the hierarchical tree of MeSH terms. We determined whether there is an interaction between health and informatics by examining the proportion of the MeSH terms included in the article that falls into either the health MeSH branch or the informatics MeSH branch. For instance, a given publication can be understood to be included in the field of MI if it is indexed with either of the following MeSH terms from 2 branches: (1) the MeSH tree parent node “Diseases [C]” or node “Health [N01.400]” or “Public Health [N06.850]” and (2) the MeSH tree parent node “Information Science [L].” This paper further demonstrates this interaction between health demand and informatics supply using visualizations such as the ternary map and sankey map.

All terms under the MeSH tree parent node “Diseases [C]” or node “Health [N01.400]” or “Public Health [N06.850]” were labelled as H. All terms under the node “Information Science [L]” were labelled as I, and all terms under node “Analytical, Diagnostic and Therapeutic Techniques, and Equipment [E]” were labelled as T.

### Research Questions

The primary goal was to measure the pattern of interactions between health demand and informatics supply in China, as well as the interplay among informatics supply, health demand, and technological applications. This paper mainly uses the United States as a comparator for investigating the development of MI in China, with a particular focus on the H-I-T interaction in each country. The research questions are as follows:

(1) What is the pattern of interactions between health demand and informatics supply as well as technological applications in China?

(2) Are informatics research efforts dealing with the major health needs that carry the greatest burden of disease in China?

(3) What is the pattern of the interplay among informatics supply, health demand, and technological applications in China for important specific areas, such as AI?

### Literature Review

#### Related Research on the Development of MI in China

To achieve universal access to medical resources, the Chinese government has put HIT as an important technical support tool for the country’s health care system. According to a longitudinal study by the China Hospital Information Management Association (CHIMA) Annual Survey, the overall adoption of electronic medical records (EMRs) in China in 2018 has surpassed that of the United States in 2015 and Germany in 2017 on average, yet with only about one-fifth of the required funding and about one-fourth of the required human resources per hospital as compared to the US HITECH project [13].

In addition, China is planning to build a regional medical consortium of hospitals based on regional HITs promoted by health information exchanges in the country. There are several studies exploring the unique contributions and characteristics of HIT development in Chinese hospitals. According to the CHIMA’s Annual Survey from 2006 to 2015, the electronic sharing of medical data among Chinese hospitals is growing rapidly. The percentage of hospitals relying solely on paperwork for data interaction declined from 43.3% in 2011 to 8.0% in 2015. There was a strong positive linear correlation between hospitals that join the consortium and the accessibility of electronic medical data exchange plan. The number of hospitals endorsing dual referral systems and appointments, allowing data to be browsed between hospitals and regional information systems, and offering remote consultation services grew to 65.0%, 61.6%, and 81.9% respectively, in 2015, compared to 18.8%, 16.8%, and 10.9% respectively, in 2011 [14]. From 2007 to 2018, 10,954 hospital Chief Information Officers across 32 administrative regions in Mainland China were interviewed in the CHIMA Annual Survey [13]. In terms of funding, the sampled hospitals’ annual HIT investment and their average investment per bed increased substantially. With regard to information system development, as of 2018, the average EMR implementation rate of the sampled hospitals exceeded the average of 2015 in US counterparts and 2017 in German counterparts (85.26% vs 83.8% and 68.4%, respectively).

However, academic research in MI lags behind HIT applications within China. Hu et al [15] revealed the notable growth of MI education, a specialty rooted in medical library and information science education, in recent years in China. Although its development has been affected by frequent name changes and an unclear identity, its success has not been entirely ignored. It is recommended that in China, (1) MI treated as a “must-have” discipline be given high priority; (2) independent, balanced degree programs be set up; (3) a specialty of “medical informatics” be established under the “medicine” category; and (4) curricula be integrated with international MI education.

Lei et al [7] argued that Chinese researchers in MI have made insufficient contributions to the global community despite China’s substantial HIT market and tremendous investments in hospital information systems. MI has traditionally been focused on medical library or bibliographic information instead of medical (hospital information or patient information) information. Its slow progress is largely due to the misdirected concentration, insufficient teaching staff who have received formal education of MI, and the incorrect positioning as an undergraduate discipline. Liu et al [16] compared MI education at the top 10 universities in 3 Asian countries. Japan and South Korea have developed modernized educational systems for MI. Universities in Mainland China offer very few curriculum systems in line with international standards and practices. Analysis of the development of MI and the current status of continuing education in China and the United States were presented from the perspective of conferences. Four MI conferences in China and 2 in the United States were conducted for both quantitative and qualitative analyses: China Medical Information Association Annual Symposium (CMIAAS), China Hospital Information Network Annual Conference (CHINC), China Health Information Technology Exchange Annual Conference (CHITEC), China Annual Proceeding of Medical Informatics (CPMI) vs the American Medical Informatics Association (AMIA) and Healthcare Information and Management Systems Society (HIMSS). CMIAAS and CPMI are mainstream academic conferences, while CHINC and CHITEC are industry conferences in China. The results showed that considering China’s economy’s scale along with the huge investment in HIT, the country is at a low level in terms of the conference output and attendee diversity [8,9]. Moreover, basic MI research funding is inadequate in China compared with the huge investments in HIT applications [7]. As such, the current development of MI in China can be characterized as “hot in industry applications and cold in academic research.”

#### Mapping Interactions Between Different Knowledge Entities Using the MeSH Tree

The MeSH thesaurus is a controlled and hierarchically organized vocabulary produced by the National Library of Medicine. It is used to index, catalog, and search biomedical and health-related information [17]. The MeSH terms are organized in a tree-like network structure consisting of 16 branches coded using A–N, V, and Z. The name of the branches are Anatomy, Organisms, Diseases, Chemicals and Drugs, Analytical, Diagnostic and Therapeutic Techniques and Equipment, Psychiatry and Psychology, Phenomena and Processes, Disciplines and Occupations, Anthropology, Education, Sociology and Social Phenomena, Technology, Industry, Agriculture, Humanities, Information Science, Named Groups, Health Care, Publication Characteristics, and Geographicals. Within each branch, MeSH terms with shorter “Tree Number” identification codes are relatively general concepts that branch out into more specific concepts. Each article in PubMed is typically assigned to several MeSH terms.

The MeSH tree is a widely recognized controlled vocabulary thesaurus for information retrieval systems [18]; it has been used to map the interactions between different knowledge entities in the biomedical and health domain.

One is to measure the translational interactions between basic research and applied research reflected by MeSH terms. Weber [19] introduced an approach to mapping PubMed articles onto a graph, called the “Triangle of Biomedicine,” by assigning articles in 3 categories (Human, Animal, and Molecular/Cellular Biology [HAC]) based on the number of MeSH terms they have that fall into each of these categories. Each publication is given a code based on whether it contains MeSH terms from that group (eg, a publication containing 1 or 10 MeSH terms from a cellular group would be given a “C”). Weber defined translation as a movement of a collection of articles, or the articles that cite those articles, toward the human corner. Based on this framework, a data science team at the US National Institutes of Health (NIH) modified the algorithm so that the HAC categories are fractionally counted, which is done for each article by dividing the number of HAC terms in each category by the total number of terms in all 3 categories [20]. In place of the binary variable Weber [19] used, NIH’s development opens up the triangle so an article can appear anywhere on it, instead of just the 7 points in the Weber triangle. Recently, Ke [21] further integrated the elements in this model. He adopted a working definition of cell- and animal-related MeSH terms as basic and human-related as applied. Ke proposed a method to place publications onto the translational spectrum, by learning embeddings of controlled vocabularies. He applied these learning methods on MeSH terms to obtain similarities between human-related terms and the rest, which in total determines the degree of basicness of the articles.

The other is measuring medical innovation through the interplay among the demand, supply, and technology in terms of MeSH terms. Several scholars have taken advantage of the fact that MeSH is organized as a hierarchical tree, and the relevant topic areas that correspond to particular MeSH nodes and their subtrees can be used to measure the process of medical innovation. Agarwal and Searls [22] were the first to conceptualize the medical innovation interaction in terms of “demand” (represented as “diseases” in MeSH terms) versus “supply” (represented as new “drugs and chemicals” in MeSH terms). Focusing on 3 main branches — “diseases,” “drugs and chemicals,” and “techniques and equipment” — Leydesdorff et al [23] used base maps and overlay techniques to investigate the translations and interactions and thus to gain a bibliometric perspective on the dynamics of medical innovations. Based on the study by Agarwal and Searls [22], Petersen et al [24] developed a triple helix model of medical innovation — supply, demand, and technological capabilities — by introducing a third branch of MeSH terms referring to “Analytical, Diagnostic and Therapeutic Techniques and Equipment” (namely, “Techniques and Equipment”), which provides yet another perspective relevant to medical innovation. Compared with only the demand and supply interactions investigated in the study by Agarwal and Searls [22], technological capabilities make it possible to observe the generated innovation in the forms of products, processes, and services.

#### HIT Innovation in Comparison With the More Well-Established Pharmaceutical Industries

HIT and evidence-based digital medicine can also be understood as a medical technology (the “supply” side) similar to drugs and devices to meet the needs of health care and disease management (the “demand” side). Worldwide, the chaotic and subpar processes and results of HIT innovation are noted in the wake of tremendous investments in capital and human resources, especially when compared with the more well-established drug and device industries [25]. “Evidence-based medicine” is best suited to deal with the uncertainty surrounding the MI and HIT applications [26]. Evidence-based MI can be defined as the “conscientious, explicit, and judicious use of the current best evidence” to support a health care decision that employs information technologies [27]. There are few studies on the application of evidence-based medicine to evaluate the effectiveness and safety of HIT and digital health interventions as well as AI algorithms on health [26,28-31]. Evidence-based MI, despite some progress, is still in the early phases of development [1]. It is the responsibility of the whole community to build evidence in MI, providing it is considered to be a scientific discipline [27]. Drug and device innovations must follow a standardized pipeline of production processes, while HIT innovations do not meet the equivalent standards. As a consequence, when it comes to producing effective and reliable products for the public, HIT lags behind the more mature drug and device industries.

As new drugs are the major source of medical innovation, informatics-related concepts and techniques are a major source of digital medical innovation. Inspired by this point, along with the aforementioned framework to measure medical innovation in the “Mapping Interactions Between Different Knowledge Entities Using the MeSH Tree” section, we suggest measuring digital medical innovations (or MI innovations or HIT innovations) by replacing “drugs and chemicals” with “information science”–related MeSH terms.

## Methods

### H-I-T Model

#### Overview

We used 3 MeSH branches as representations of *H*ealth demand, *I*nformatics supply, and *T*echnological applications (the H-I-T model) and used their co-occurrences to measure the digital medical innovation process in China. The detailed definition of HIT is as follows. For “H,” the entire “Diseases [C]” branch as well as 2 subbranches (ie, “Health [N01.400]” and “Public Health [N06.850]”) are regarded as a representation of health demand for HIT innovations. “Health [N01.400]” and “Public Health [N06.850]” are under the branches “Population Characteristics [N01]” and “Environmental and Public Health [N06],” respectively — with the top root “Health Care [N].” So, we use 2 MeSH terms, “health” and “public health,” to represent the population health demand and the “diseases category” MeSH terms to indicate the individual health demand (specific disease management).

For “I,” the “Information Science [L]” branch is a representation of the supply side in terms of informatics concepts and techniques.

For “T,” the “Analytic, Diagnostic, and Therapeutic Techniques and Equipment [E]” branch is a representation of state-of-the-art technological applications, namely the functions to be realized by informatics (eg, diagnosis, therapeutics, surgical procedures, investigative techniques, equipment, and supplies).

In the H-I-T model, every related article can be classified as health demand (H), informatics supply (I), technological applications (T), or a combination of these 3 using the MeSH terms and HIT score. MeSH terms are arranged in an alphabetical and hierarchical structure from the most general level to the narrowest level. Table 1 shows the branches of MeSH terms used in distinguishing the H-I-T classification. Note that since the MeSH term “Public Health” has another tree number H02.403.720 (branch of medicine concerned with the prevention and control of disease and disability and the promotion of physical and mental health of the population on the international, national, state, or municipal level), terms under this branch are also included in the health demand (H) category. These terms include Epidemiology, Molecular Epidemiology, Pharmacoepidemiology, Preventive Medicine, Environmental Medicine, Occupational Medicine, and Preventive Psychiatry.

**Table 1:** Medical Subject Heading (MeSH) terms used in each health demand, information supply, technological applications (H-I-T) category

H-I-T category	MeSH branches	Number of terms
Health demand (H)	Diseases [C], Health [N01.400], Public Health [N06.850]	5331
Informatics supply (I)	Information Science [L]	419
Technological applications (T)	Analytical, Diagnostic and Therapeutic Techniques, and Equipment [E]	2985

It is noted that for each publication, only a MeSH major topic (ie, MeSH [primary] terms) are used in our data collection and computation. In PubMed publications, a MeSH term that is one of the main topics discussed in the article is denoted by an asterisk(*) on the MeSH term or MeSH/Subheading combination and is referred to as a MeSH major topic. The major topic can reveal the most essential research content of an article.

#### Mathematical Description of the H-I-T Model

**Figure 1:**
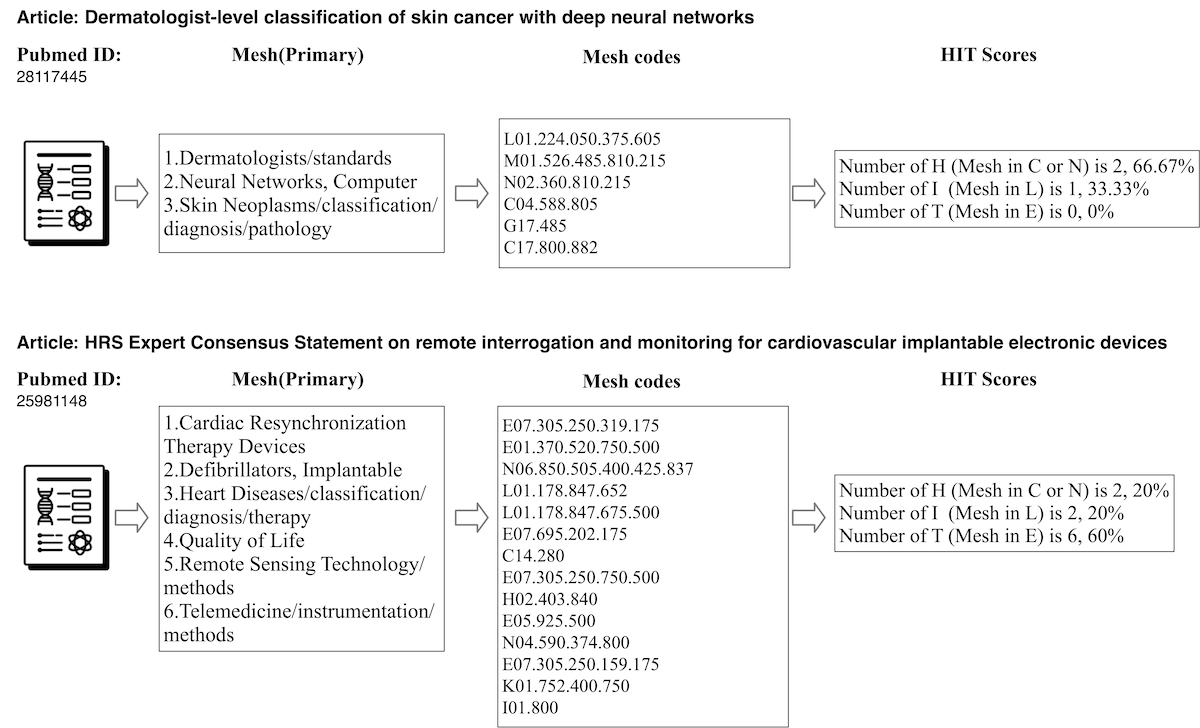
The calculation process of health demand, informatics supply, technological applications (H-I-T) scores for 2 example articles.

The classification algorithm calculates the percentage for each category by dividing the number of H-I-T MeSH (primary) terms in each category by the total number of terms in all 3 categories. Figure 1 shows 2 examples of calculating HIT scores. The first article with PMID 28117445 was tagged with 3 MeSH (primary) terms. It is noted that 1 MeSH term may belong to 2 or more branches and have 2 or more MeSH codes. In this situation, we marked each MeSH code once. Now, 3 terms became 6 MeSH codes; the codes beginning with C or N06.850 or N01.400 were classified as “H.” The codes beginning with L were classified as “I.” The codes beginning with E were classified as “T.” The final HIT scores were calculated using the codes belonging to the 3 H-I-T categories only, for instance, as indicated in Figure 1 with a total of 3 H-I-T MeSH terms: 2 for H, 1 for I, and 0 for T. The final H-I-T scores for this article are H=2/3, I=1/3, T=0, with only the linkages between H and I and none with T.

The H-I-T scores for the second article with PMID 25981148 were also calculated using the same algorithm. It has a total of 10 H-I-T MeSH terms: 2 for H, 2 for I, and 6 for T. The techniques and equipment (“Cardiac Resynchronization Therapy Devices,” “Defibrillators, Implantable,” and “Remote Sensing Technology”) have linked the health demand (“Heart Diseases,” “Quality of Life”) with the informatics supply (“Telemedicine”). Without such techniques and equipment as the “Remote Sensing Technology,” it is hard to apply telemedicine to heart disease care.

#### Visualization of the H-I-T Model

**Figure 2:**
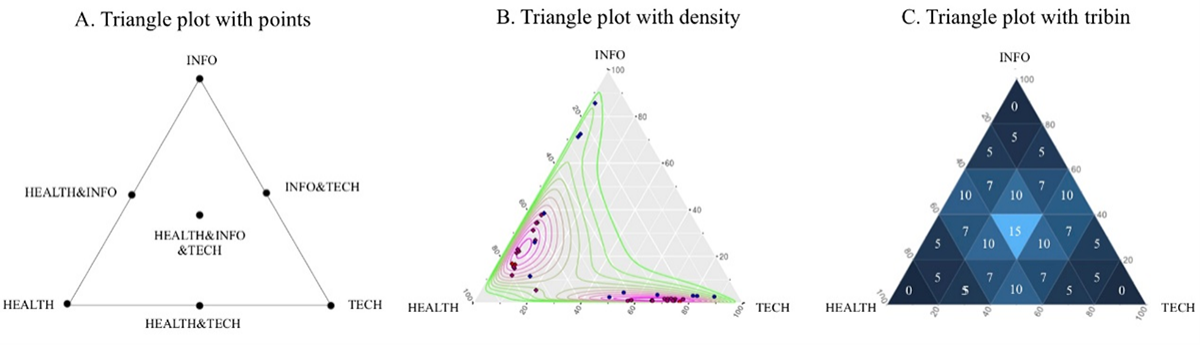
Three ways to display the health demand, informatics supply, technological applications (H-I-T) triangle: (A) triangle plot with points only on the vertex, middle of the edges, and center; (B) triangle plot with points and a density contour; (C) triangle plot with tribins.

To maximize the utility of the H-I-T model, we adapted Weber's Triangle of Biomedicine [19] to show the composition of systems composed of H-I-T. Each of the 3 apexes represents health demand (H), informatics supply (I), and technological applications (T). If an article contains a 100% H or I or T, it will be placed at one of the vertices of the triangle as a dot. If an article contains a 50% H and 50% I, then it will be placed in the middle of the left edge of the triangle, as shown in Figure 2A. If an article contains at least 33% H/I/T, then it will be placed in the center of the triangle, and so on.

Usually, there are only 5 to 10 MeSH (primary) terms in a paper, and the percentage repetition rate will be high when calculating H-I-T scores for each article. There will be a large number of points overlapping on the triangle graph, and the points themselves lose their meaning due to visual clutter. Often, scholars use density contours in triangles (Figure 2B) to improve visualization, but those density markers alone are still difficult to observe quickly by the human eye. This paper further improves on the display details of the triangle by dividing the entire triangle into many mini tribins. We cut the whole triangle into *N* equal parts in 3 directions, and then the N*N small tribins appear within the original large triangle. It enables us to bin points in small triangular areas; the number of points in each small area can be counted. With large datasets, we are able to display the counted number and color on tribins together (Figure 2C). Perhaps it can add more richness to triangle diagrams and thus enhance the visualization of the HIT triangle diagram. The triangle diagrams in this paper were implemented by using ggtern library in R [32].

### Mapping ICD-10 to MeSH Terms

To approximate the extent of health needs, we use the World Health Organization (WHO) Global Burden of Disease (GBD) survey as useful information. The WHO provides the corresponding codes of the International Classification of Diseases (ICD-10) in which each of the aforementioned diseases is classified.

Most recently, Yegros et al [33] matched WHO ICD-10 with MeSH terms in a corresponding table to find whether research efforts address global health needs. We reviewed this correspondence table again and used it to map the correlation between disease burden and rates of informatics-related publications for China. To link publications to diseases, we used not only the MeSH terms assigned to ICD-10 codes but also all MeSH terms located beneath them in the MeSH tree. This, for instance, enabled us to assign publications with the MeSH term “Diabetic Nephropathies” to the disease “Kidney Diseases” even if the MeSH term “Kidney Diseases” was not assigned to these publications. In fact, the term “Diabetic Nephropathies” is the subordinate concept of the term “Kidney Diseases.” Table 2 shows the correspondence table between ICD-10 and MeSH for specific cardiovascular diseases.

**Table 2:** Correspondence table between International Classification of Disease (ICD)-10 and Medical Subject Headings (MeSH) for 2 specific cardiovascular diseases.

Cardiovascular disease	ICD-10 code	Matched		Excluded	
		MeSH Tree Number	MeSH terms	MeSH Tree Number	MeSH terms
Hypertensive heart disease	I10-I15	C14.907.489	Hypertension	C13.703.395; C14.907.489.480	Hypertension, Pregnancy-Induced
Ischemic heart disease	I20-I25	C14.280.647; C14.907.585	Myocardial Ischemia	N/A^a^	N/A

^a^N/A: not applicable.

### Data Collection

In order to systematically collect publications relating to informatics supply and health demand, here, we use a new approach to collect publications that provide a broad interpretation of MI using the hierarchical tree of MeSH 2020 terms. A given publication can be understood to be included in the field of MI if it is indexed with both of the following MeSH (primary) terms from each of the 2 branches: (1) the MeSH tree parent node “Diseases [C]” or node “Health [N01.400]” or “Public Health [N06.850]” and (2) the MeSH tree parent node “Information Science [L].” Note that we restrict our analysis to the “Major Topic Headings” for each article, which are indicated in each PubMed article page by an asterisk * next to the MeSH term; these MeSH (primary) terms are sufficient to identify the article’s core content.

A total of 213,215 publications during 2010-2020 (till June, 30 2020) were initially collected from MEDLINE using the co-occurrences of the 2 branched MeSH (primary) terms. We excluded publications indexed by such MeSH (primary) terms as (1) “Systematic Reviews as Topic” and (2) “Meta-analysis as Topic.” While located within the parent MeSH tree of “Information Science” and “Public Health,” they do not reflect the informatics supply and health demand, respectively, but represent a secondary research approach. Other exclusion criteria are given to such publications with publication types as Review and Retracted publications, as well as with the keyword “bibliometric” occurring in the title. This process leaves 194,567 global publications for the following analysis.

## Results

### Overall Results

**Figure 3:**
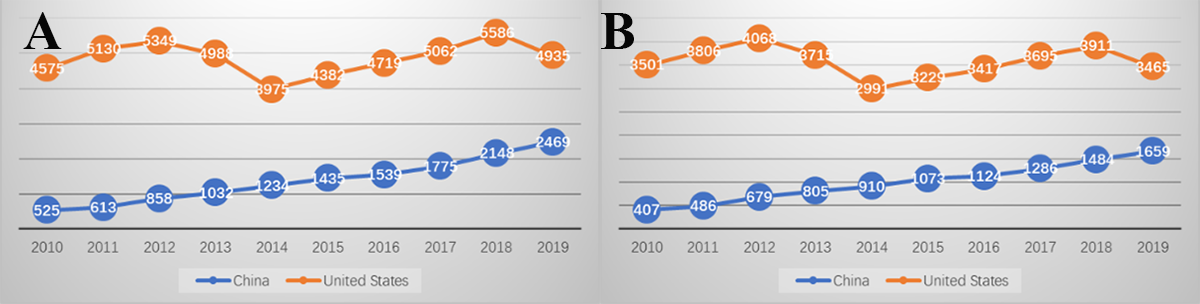
Number of (A) health demand and informatics supply (H-I) and (B) health demand, informatics supply, and technological applications (H-I-T) publications for China and the United States.

The United States ranked first based on the number of publications, accounting for one-quarter of the world’s total publications. China ranked second, with the number of publications basically equal to the third, the United Kingdom. Compared with the United States and United Kingdom, China’s publications increased rapidly since 2010, when the Chinese government launched health reforms for the second time and invested significant funding in HIT (Figure 3, Table 3).

It is noted that of all MI papers published by China, there are 1083 in the Chinese language, published in MEDLINE-indexed Chinese journals, such as *Sheng Wu Yi Xue Gong Cheng Xue Za Zhi* (N=126), *Nan Fang Yi Ke Da Xue Xue Bao* (N=80), and *Zhonghua Liu Xing Bing Xue Za Zhi* (N=77; Table 3). It reflected the interactions among MI with biomedical engineering as well as epidemiology, which is an important subdiscipline of public health and preventive medicine in China. We first performed an exploratory data analysis and found that the pattern of interactions between health demand and informatics supply for the United Kingdom and China is similar. There is a significant difference between the United States and United Kingom/China. In the following section, we will primarily compare China with the United States and answer the aforementioned research questions.

**Table 3:** Distribution of global publications on medical informatics (n=194,567).

Ranking	Country/territory	Number of publications	%
1	United States	49,353	25.4
2	China	14,105 (1083 in Chinese, 13,022 in English)	7.2
3	United Kingdom	13,783	7.1
4	Germany	9469	4.9
5	Canada	8326	4.3
6	Australia	7110	3.7
7	Italy	6644	3.4
8	Japan	6443	3.3
9	France	6037	3.1
10	The Netherlands	5596	2.9

### Interactions Between Health Demand, Informatics Supply, and Technological Applications

#### Overall H-I-T Interactions

First, we calculated the average H-I-T scores of publications for the world, the United States, and China. As shown in Table 4, we found that if we only count the average scores, the H-I-T scores for China, the United States, and the world are very similar. In general, the H score is higher than the I and T scores, indicating that the number of H-related MeSH major topic terms is more than those of I- and T-related topics. In other words, this research tends to be health demand–oriented. Then, we counted the H-I and H-I-T interacting publications for China and the United States (Figure 3). Compared with the fluctuating trends for the United States, the interactions of both H-I and H-I-T for China show a notable increasing trend.

**Figure 4:**
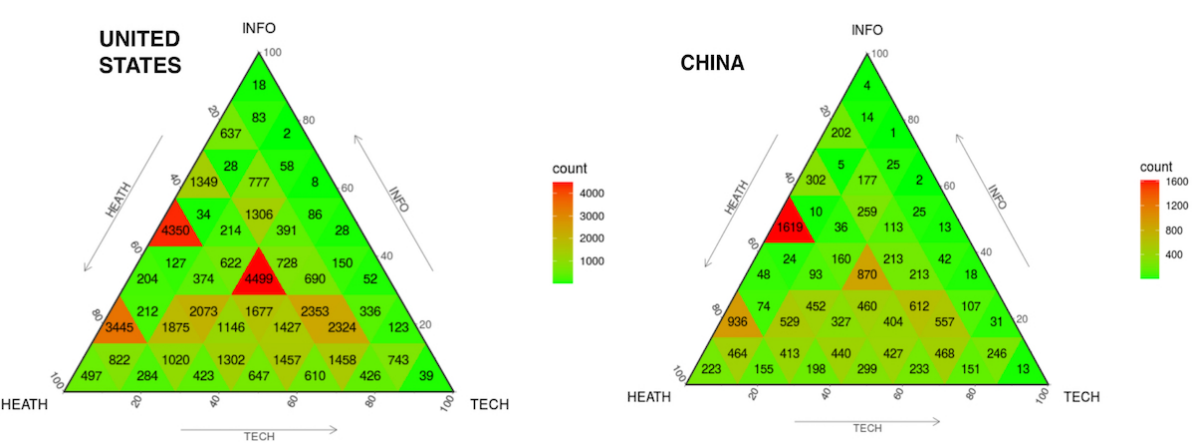
Overall layout of publications in the health demand, informatics supply, and technological application (H-I-T) triangle for the United States and China.

Next, we mapped publications from China and the United States on the H-I-T triangle graphs based on the H-I-T model, as shown in Figure 4. Since the search strategy was that any given paper will definitely contain the H and I MeSH terms, we would naturally assume that most articles on the triangle would tend towards the edges H and I. However, the distribution of the United States’ publications was slightly unexpected. In the US triangle diagram, the reddest subtriangle is in the center (N=4499) instead of the edges of H and I (N=4350). The distribution of Chinese publications in the triangle is quite different from that of the United States; they are much less prone to the T side of the triangle. Most articles from China are located at the edges of H and I. In the just-centered subtriangle, the number of articles with the same percentages of H, I, and T MeSH primary terms (each category accounts for one-third) is much less for China (N=870) than the United States (N=4499). This shows that, while China’s existing techniques and equipment have been established to link informatics supply and health demand, the informatics research efforts in the United States have provided a stronger H-I-T link than China.

**Table 4:** The average health demand, information supply, and technological application (H-I-T) scores for the world, the United States, and China.

Country/territory	H	I	T
United States	0.434	0.280	0.287
Global	0.436	0.277	0.288
China	0.450	0.268	0.282

#### Detailed H-I-T Interactions

Such differences between China and the United States are also observed when the detailed H-I-T interactions are considered. We use the first level of disease classification in the MeSH tree and combine “Health [N01.400]” and “Public Health [N06.850]” as “population health.” The first-level concepts in the “Information Science” branch and “Analytic, Diagnostic, and Therapeutic Techniques and Equipment” branch are used to map the interactions between informatics supply, technological applications, and health demand, which is represented by various specific diseases and population health.

**Figure 5:**
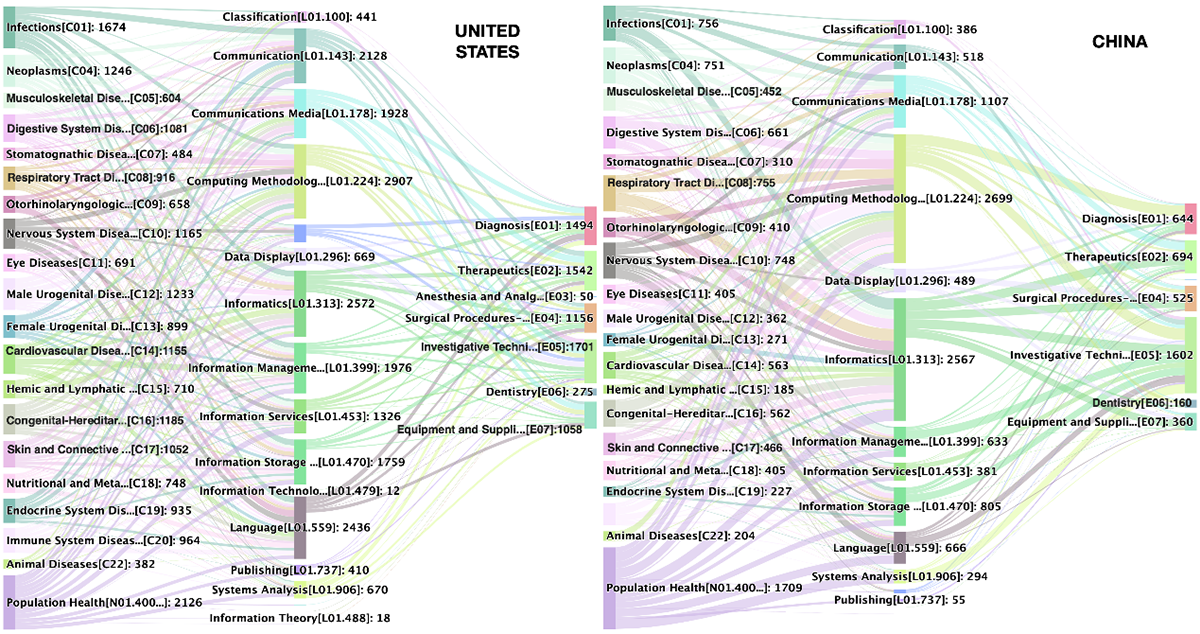
Detailed profile of health demand, informatics supply, and technological application (H-I-T) interactions for the United States and China.

Figure 5 depicts the detailed profile of the H-I-T interactions. In general, such interactions in China are relatively weaker compared with those in the United States. According to Figure 5, whether it comes to diseases, informatics, or technology applications, the United States is more balanced, while China is more concentrated. Computing methodologies, informatics, and communications media (such as social media and the internet) constitute the majority of the informatics domain and are the 3 most common HITs used for resolving the health and disease problems in China. In China, social media, the internet, and other forms of communication media are not only used to solve public health problems but also applied to specific disease problems, especially infections diseases, cardiovascular diseases, respiratory tract diseases, neoplasms, nervous system diseases, and many other chronic diseases. The State Council has released a medium to long-term plan (2017-2025) on the prevention and treatment of chronic diseases and emphasized the roles of internet+health in promoting health. Thus, social media and other internet media are extremely important for health promotion and self-care among patients and their family members and caregivers.

As is shown in Figure 5, population health is the largest informatics research target for both the United States and China, since public MI has been an established research area for both of them. In fact, the term “public health informatics” was introduced into the MeSH tree in 2003 and defined as “the systematic application of information and computer sciences to public health practice, research, and learning.” But there were some differences in the health demand domains interacting with informatics between China and the United States. For China, the major diseases supported by informatics research efforts include nervous system diseases, neoplasms, digestive system diseases, cardiovascular diseases, and respiratory tract diseases. The types of diseases that are most interactive in China are ranked rather low in the United States. Such cases were observed for male urogenital diseases, immune system diseases, and nutritional and metabolic diseases. In other words, China and the United States are using similar HITs to solve their own health problems.

### Informatics Research Efforts vs Burden of Disease in China

The question to be discussed in this subsection is whether China informatics research efforts are dealing with the major health needs, measured by the burden of disease. The Years of Life Lost was used, and Disability-Adjusted Life Years (DALYs) were provided by the WHO as a proxy of the burden of disease. Although DALYs are not free of limitations, they are one of the most established proxies of disease burden. We identified informatics-related publications related to a selection of the diseases considered in the WHO GBD estimates for the year 2016 [34], which is much closer to the publication search window used in this paper. Combined with the rankings of the top 25 leading causes of DALYs in China during 1990-2017 [35], we considered the 24 most specific diseases with high DALYs in China in the “Communicable, maternal, perinatal and nutritional conditions” and “Noncommunicable diseases” groups. We did not consider diseases in the “Injuries” group since it is difficult to match them with MeSH terms, although “road injuries” ranked fifth in the leading causes of DALYs in 2017.

**Figure 6:**
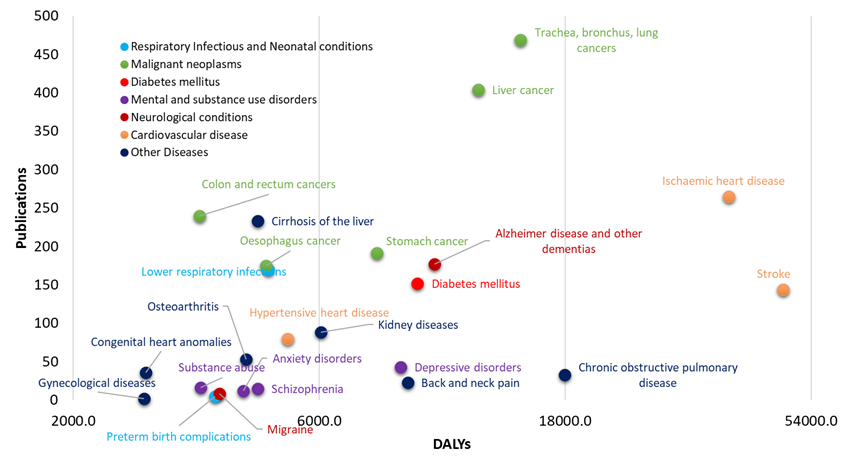
Top 24 disease-related Disability-Adjusted Life Years (DALYS) versus informatics-related publications in China.

According to Figure 6, generally, there is a positive correlation between the burden of disease and the oriented informatics research publications, when analyzed by specific therapeutic areas classified by the WHO. The linear regression results showed y = 0.0079x, R² = 0.4103. Two major cardiovascular diseases, stroke and ischemic heart disease, are leading causes of DALYs in China and in recent years, have attracted a considerable amount of information science research. For several major malignant neoplasms, such as lung cancer, liver cancer, stomach cancer, esophagus cancer, and colon and rectum cancers, although their DALYs vary largely, research has attracted the most informatics research efforts. Among the top 24 diseases related to DALYs in China, there is a gap between the burden of disease and informatics research efforts for chronic obstructive pulmonary disease, back and neck pain, and depressive disorders, which have a higher burden yet lower informatics research efforts. Overall, among mental disorders, beside depressive disorders, schizophrenia, substance abuse, and anxiety disorders have disproportionate informatics research efforts to their disease burden.

### Interactions Between Health Demand, AI Supply, and Technological Applications

**Figure 7:**
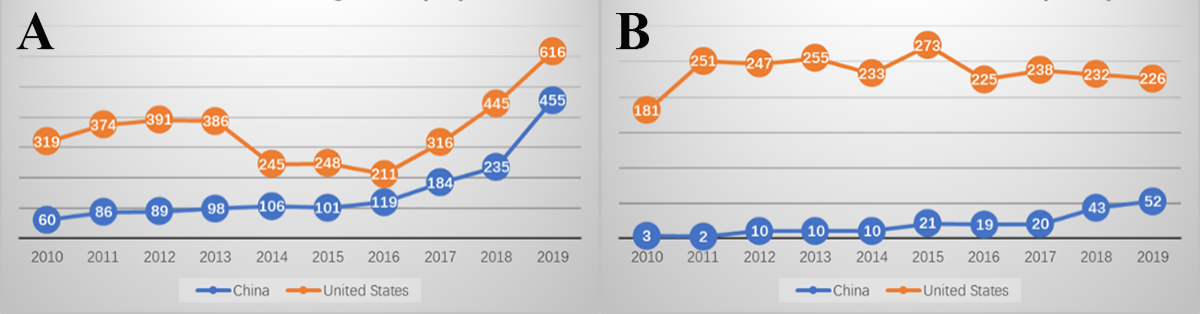
Publications about (A) artificial intelligence and (B) electronic health records for the United States and China.

The “computing methodologies” represent the largest part of information science research for both the United States and China, of which AI is the most dominant area. We use the “Artificial Intelligence” MeSH terms and all terms beneath it in the MeSH tree to construct AI sub-datasets. According to Table 5, the proportion of AI-specific publications across all publications linking health demand and information science supply is significantly higher for China (11.3%) than the global average level (6.8%) and that in the United States (7.4%). The number of AI publications has grown rapidly, especially since 2016 (Figure 7). In the MeSH tree, “Artificial Intelligence” is defined as the theory and development of computer systems that perform tasks that normally require human intelligence. Such tasks may include speech recognition, learning; visual perception; mathematical computing; reasoning, problem solving, decision making, and the translation of language. It has 8 subbranches: (1) Computer Heuristics, (2) Expert Systems, (3) Fuzzy Logic, (4) Knowledge Bases (ie, Biological Ontologies), (5) Machine Learning, (6) Natural Language Processing, (7) Computer Neural Networks, and (8) Robotics.

**Figure 8:**
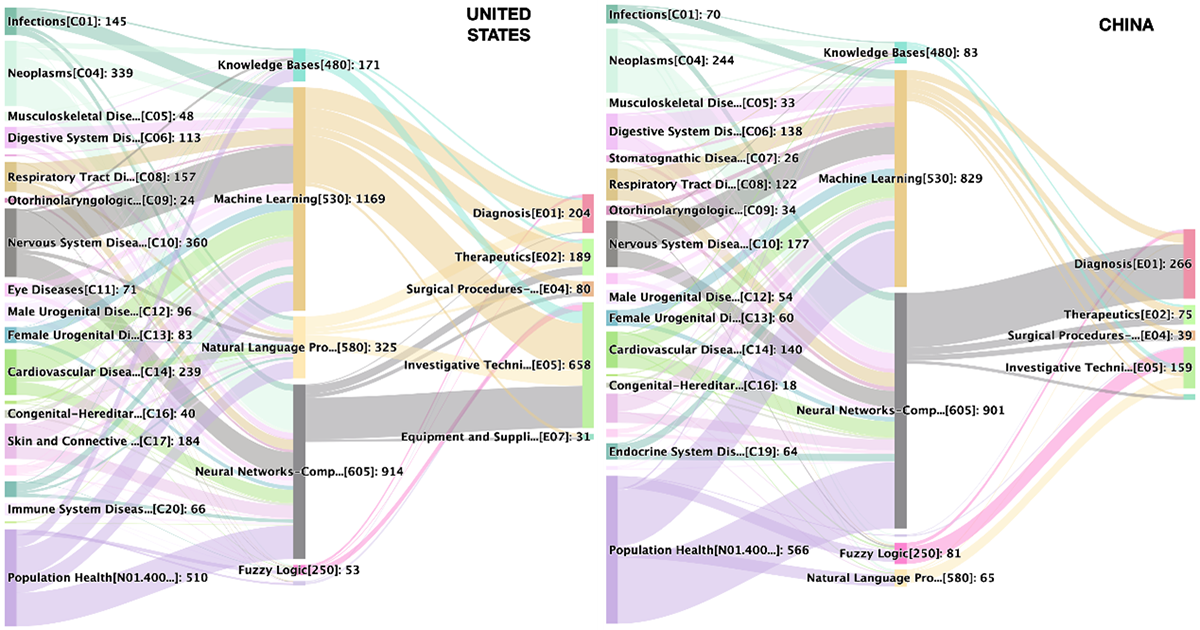
Comparison of health demand, AI supply, and technological application (H-AI-T) interactions for the United States and China.

As a subfield of information science, AI and related technologies are increasingly prevalent in medical research and are beginning to be applied to health care and medical research. In this section, we specifically analyzed and discussed the H-I-T interactions in AI-related research publications. Figure 8 shows the H-I-T interactions in AI research in China and the United States, and the secondary MeSH terms under MeSH topic “Artificial Intelligence [L01.224.050.375]” were selected to calculate co-occurrence relationships between health demand, AI supply, and technology applications.

As to the connection between health demand and AI supply, the most focused domain of health demand is population health; the most concentrated AI concepts are computer neural networks and machine learning; and the most extensive technology applications are investigative techniques, diagnosis techniques, and therapeutic techniques. Investigative techniques are commonly used in preclinical and clinical research, epidemiology, chemistry, immunology, and genetics, among others. The investigative techniques do not include techniques specifically applied to diagnosis, therapeutics, anesthesia and analgesia, surgical procedures, surgical, and dentistry. After a detailed analysis of the specific topics under “Investigative techniques,” we found the most focused topics are “observation,” “research design,” and “epidemiologic methods” and “models, theoretical.” We concluded the linkage “Population health—machine learning (including deep learning such as neural networks)—Investigative techniques” shows hot topics such as machine learning–enabled clinical research and disease prediction models based on real-world data. Now, we turn from “population health” to specific diseases. Both countries have used AI technologies for research on all diseases. Among those, nervous system diseases and neoplasms are the most focused AI-targeted diseases for the United States and China, respectively. Diseases that very rarely use AI include infections, otorhinolaryngologic diseases, immune system diseases, and hemic and lymphatic diseases.

On the informatics supply side, machine learning and neural networks are the most commonly used techniques in both Chinese and US publications. In the United States, “machine learning” and “computer neural networks” co-occurred 1169 and 914 times, respectively, with health demand–related terms. China, on the other hand, was counted 829 and 901 times, respectively. In health AI research by US scholars, “computer neural network” technologies have been used mainly in clinical research and real-world studies, with 248 occurrences, whereas it is rarely used for diagnosis [E01], with only 5 occurrences. However, in Chinese scholars’ research, “computer neural network” technology has been mainly supported for “Diagnosis” [E01] with 209 co-occurrences.

Furthermore, another significant difference observed between health AI research by Chinese and US scholars is the use of natural language processing technology. Natural language processing co-occurred 325 times with healthy demand for the articles by US scholars, but only 65 times for China. As it is indicated in the Sankey diagram (Figure 8), the most common use of natural language processing techniques in the United States focused on “Population Health,” followed by cardiovascular disease, nervous system disease, and many other specific diseases.

The phenomenon that natural language processing–related research seems a gap or a weak point for China may be due to the relative lack of research on electronic health records (EHRs) in China. The MeSH term “Electronic Health Records” is a standardized term that unifies synonyms such as Computerized Medical Records and EMR. It is defined as media that facilitate transportability of pertinent information concerning a patient’s illness across varying providers and geographic locations. Some versions include direct linkages to online consumer health information that is relevant to the health conditions and treatments related to a specific patient. While continuously increasing (Figure 7), China has insufficient research publications as to its overall health information research publications on EHR (193, 1.4%), compared with the world average (3.3%) and the United States (4.8%).

**Table 5:** Comparison of the number of publications between health demand and artificial intelligence (AI) supply.

Country/territory	Number of AI publications	Number of all H-I^a^–interacted publications	Percentage (%)
Global	13,258	149,567	6.8
China	1592	14,105	11.3
United States	3628	49,353	7.4

^a^H-I: health-related MeSH (Medical Subject Headings) terms co-occurred with information-science-related MeSH terms.

## Discussion

### Interactions Between Health Demand, Informatics Supply, and Technological Applications

Informatics uses the synergistic “bridging” of electronic data to benefit individual diseases and population health. There has been a consistent increase in the number of publications tagged with both health-related and information science–related MeSH terms since 2010 in China. This is in accordance with the observation that “a significant upward trend particularly after 2011 in the number of articles by Chinese academics in MI based on their publications in 18 international specialty journals” [12]. They also concluded that the global influence of Chinese scholars is growing worldwide; they are making increasingly conscious efforts to enhance their collaborative relationships with international researchers. In their contribution, the hottest and emerging technological fields in MI were examined, such as EMRs, AI, and image processing, whereas the functions these informatics technologies have realized, such as in supporting diagnosis and therapeutics of diseases, and the health needs they are used to address have not been investigated. The focus of our paper is the interplay between knowledge entities through the co-occurrence of the 3 dimensions of health-, information-, and technology-related terms, instead of macrobibliometric analysis. Our results suggest that the interactions between health demand and informatics supply as well as technological applications in China are showing significant growth. Among the top 3 countries with the highest number of publications, the number of H-I-T publications in China is growing the fastest. In our analysis, population health or public health is the area of greatest demand that interacts with informatics for both the United States and China. Population health can have the most impact on health informatics research. Recently, Bhattarai et al [10] investigated how information and communications technologies (ICTs) were applied to public health and found that “communicable disease monitoring,” “public health policy and research,” and “public health awareness” are the most common public health domains interacting with ICTs. One of the limitations of their study is that, by only using one MeSH tag as a selection criterion, publications without the “public medical informatics” MeSH term were excluded from their dataset. Our research avoids such a limitation.

Despite the increasing output of academic research, the overall interaction between health demand, informatics supply, and technological applications in China is weaker than that in the United States. To some extent, this has affected the technological transfer of HITs into products and ultimately had an impact on the development of evidence-based digital medicine. The effectiveness and safety of HIT must be evaluated scientifically before it can be used by doctors, patients, and consumers. For example, Bhattarai et al [10] reported that inconsistent results exist regarding the validity of most of the informatics indicators when various predictors are used for disease surveillance and emergency monitoring, such as syndromic surveillance, dispatch calls, over-the-counter drug sales, and school absenteeism. They suggest additional studies should be conducted to further investigate the validity of such predictions. Whereas, for evidence-based medicine, there are clear guidelines on the development and assessment of the effectiveness of biomedical or behavioral health interventions, there is a scarcity of guidelines for the systematic development and assessment of medical and health informatics, and corresponding research has just begun [25].

### Informatics Research to be Focused on the Greatest Burden of Diseases or Where It Can Have the Most Impact

We are not prepared to emphasize the idea that informatics research efforts must be proportional to the burden of disease. We think it is not contradictory that informatics research should be focused on the greatest burden of diseases or where it can have the most impact. In fact, we have taken into account these 2 viewpoints. The overall results of both the H-I-T interactions and the H-AI-T interactions indicate that population health or public health is the area with the greatest demand that interacts with informatics supply and technological applications for both the United States and China. We think population health can have the greatest impact on health informatics research. Population health and specific disease management are 2 major demanding health domains. While the extent of demand for specific diseases is easy to measure (eg, using burden of disease data), the extent of the demand for population health is not easily quantified since it is independent of diseases.

**Figure 9:**
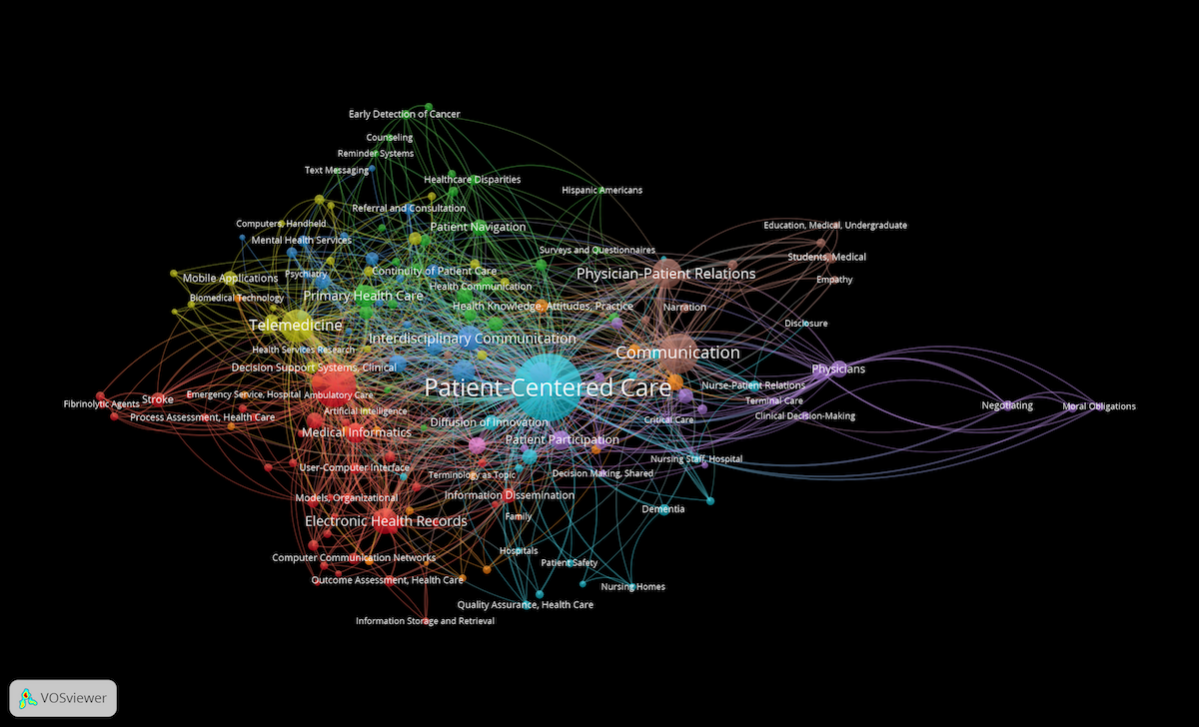
The co-occurrence clusters of MeSH Major Topics in “Patient-Centered Care”–related informatics research publications.

We used 2 MeSH terms, “health” and “public health,” to represent population health demand and the “diseases category” MeSH terms to indicate the individual health demand for specific diseases. We noticed that there are 2 MeSH terms, “Delivery of Health Care, Integrated” and “Patient-Centered Care,” that are related to health demands independent of specific diseases. The term “Delivery of Health Care, Integrated” is a health care system that combines physicians, hospitals, and other medical services with a health plan to provide the complete spectrum of medical care for its customers. In a fully integrated system, the 3 key elements — physicians, hospital, and health plan membership — are in balance in terms of matching medical resources with the needs of purchasers and patients. The term “Patient-Centered Care” represents a design of patient care wherein institutional resources and personnel are organized around patients rather than around specialized departments. Unfortunately, these terms are not included in our conceptual framework and data collection. Here, we tried to determine the research landscape between “Patient-Centered Care”/“Delivery of Health Care, Integrated” and “Information Science.” Using such a search strategy, “(“Patient-Centered Care” OR “Delivery of Health Care, Integrated”) AND (“information science category”),” with the search field “MeSH Major Topic” from the MEDLINE database, we collected 2071 publications published between 2010 and March 15, 2021. We mapped the co-occurrence clusters of MeSH Major Topics with at least tagged by 10 publications and found that “Patient-Centered Care” tended to link with nursing practices, such as “nursing staff, hospital,” “nursing homes,” “family,” “nurse-patient relations,” “patient participation,” and “patient safety” (Figure 9).

Recently, there was an urgent discussion about a shift towards integrated patient-centered models of care [36]. We may conclude from these points that the focus of nursing informatics is oriented around patient-centered care, while MI is disease-oriented, and health informatics is population health–oriented. According to a WHO report published in 2015 [37], integrated people-centered health services mean putting the comprehensive needs of people and communities, not only diseases, at the center of health systems and empowering people to have a more active role in their own health. Traditional models of care, providing hyperspecialized, program-specific delivery to narrowly defined patient cohorts (eg, cardiac programs, diabetes programs), are unable to support integrated complex needs in a manner that achieves optimal outcomes. The focus, therefore, needs to return to the holistic treatment of the whole person, to be culturally and socially sensitive to individual needs, and where appropriate, to include individual, family group, or community models of care. Blending informatics expertise with nursing’s unique perspective on holistic health care ideally situates the profession to inform the integration of emerging models of care in a digital environment. We think that is a great opportunity for the development of nursing informatics.

### Interactions Between Health Demand, AI Supply, and Technological Applications

In our study, “computer neural networks” was one of the hottest informatics techniques in health AI research. China, in general, has only less than half as many health AI publications as the United States, yet has almost the same number of computer neural network publications as the United States. Research from Chinese scholars in the field of health AI primarily focuses on deep learning and is more likely to apply complex deep learning models (such as deep neural networks) to health and medical diagnoses. This echoes the findings of a recent systematic review that compared the performance of diagnostic deep learning algorithms for medical imaging with that of expert clinicians [29]. Of the 10 randomized trial registrations for deep learning algorithms that were ultimately included, 8 were from China, and 1 was from the United States. Two trials have been completed, both from China, and their results were published in 2019. For the 81 nonrandomized studies that were included, the top 4 countries were as follows: United States (24/81, 30%), China (14/81, 17%), South Korea (12/81, 15%), and Japan (9/81, 11%). Chinese scholars are very active in diagnostic deep learning algorithms for medical imaging. AI-powered imaging became the most mature field within the intelligence and medical science industry, boasting a large market scale, substantial revenue earnings, and a favorable financing environment.

It should be noted that natural language processing is not a fully studied domain in health AI research in terms of H-I-T interactions. After reading the titles of publications, we found that almost all of the US studies in this dataset that used natural language processing were related to EHRs. This is yet further evidence that the level of openness of EMR data in Chinese significantly limits the opportunities for scholars to uncover its value.

The data show that EHRs are not fully studied, especially in China. This coincides with the following evidence from the United States as well as China. Through in-depth qualitative interviews across the United States, Sheikh and colleagues [38] investigated how to improve patient care and population health with HITs and how to reduce health care expenditure. Yet, they found that the following concerns persisted under existing systems: poor usability of EHRs, limited ability to support multidisciplinary care, and major difficulties with using the health information exchange systems. On the other hand, Zhang and colleagues [39] explored the health applications for big data in China. Although more than 90% of Chinese hospitals use EMRs, sharing data is still difficult with hospital-based systems because they are developed by more than 300 vendors using different data standards. The investigation in the United States revealed that despite the government’s substantial investment in information systems, there were barriers to integrated care due to system fragmentation. In China, the National Health Commission hosts the EHRs, the National Healthcare Security Administration hosts insurance claims data, and each hospital has set up a unique medical record system, but none of which are interoperable [5]. As such, it is critical to integrate the myriad of electronic health, medical, and claims records into a unified information system at all provider levels. Even with these challenges, the Chinese government vigorously promoted the development of big data and its application in the health and medical fields. China’s State Council has announced that it will establish a national and provincial integrated population health information platform to facilitate data sharing, clinical research, and public health initiatives in the country.

The limitations of this study include that (1) only one database (ie, MEDLINE) was searched; (2) by using the combination of 2 branches of MeSH tags as a selection criterion, publications without or with inaccurately indexed MeSH terms could not be collected; and (3) the mappings of H-I-T interactions are probably not sufficient. A much more complicated and granular MeSH-based classification technique could have been developed. However, keeping the definition of the 3 H-I-T areas simple did not seem to limit our analysis, but rather it made the results easier to interpret. In addition, only using the MeSH Major Topics seems too strict, yet this approach ensures the core content of one given publication. In many countries, researchers need funding, and these will often be determined by research funding bodies or industry, which will determine research thrust and publications. In addition, in some countries where many applied research and developments are within publicly funded health institutions, publication is not prioritized even where there is innovation; in countries where HIT is competitively, commercially produced, research sponsors may limit publication to avoid what is seen as loss of commercial advantage.

### Conclusions

This study proposed a new approach to mapping the interplay between different knowledge entities by using the tree structure of MeSH to gain insights into the interactions between health demand, informatics supply, and technology applications in China. This method can help to collect publication data with a broader interpretation of medical and health informatics and may also be applied to other interdisciplinary fields, such as medical physics, medical engineering, and medical social sciences.

China's emphasis on medical information technologies or HITs began with the new round of health care reform in 2009. Since then, medical and MI research in China has grown very fast, and the number of publications has exceeded that of the United Kingdom. The United States shows a relatively stable publication trend. While China has made these advances, some institutional and academic gaps still need to be filled in order to fully utilize the advantage of informatics in medical research and health care services. The following observations made throughout the analysis are described in the following paragraphs.

The interplay between health demand and informatics supply for China is slightly sparse, and the interactions between them were mostly observed in cardiovascular diseases, nervous system diseases, neoplasms, and population health, which are studied more with the help of computational methodologies and informatics techniques. Other techniques, such as social media, the internet, and other communication media are mainly used to solve public health problems and are rarely used in other disease research in the United States. While technological applications (T) have been established to link informatics supply (I) and health demand (H), the H-I-T linkages in informatics research in China are weaker than research in the United States. It is suggested that technological transfers, namely the functionality to be realized by medical/health informatics (eg, diagnosis, therapeutics, surgical procedures, laboratory testing techniques, and equipment and supplies) should be strengthened.

There is a positive correlation between the burden of diseases in China and the informatics research efforts for diseases. The major diseases targeted by informatics research efforts are cardiovascular diseases, neoplasms, and respiratory tract diseases, which differ in profile from those in US populations. China and the United States are using similar HITs to solve the different health needs in their respective countries.

China is unbalanced in its use of a combination of information science and medical and health sciences. The overall H-I interactions in China are sparse, focusing on several major diseases and 2 major informatics techniques. Research on EHRs combined with natural language processing should also be strengthened in China to improve the real-world applications of HITs and big data in health and medicine in the future.

All data used to calculate the HIT scores are stored in the Science Data Bank, which includes MeSH terms, MeSH tree list, and the paper list with HIT scores [40].

## Abbreviations

AI: artificial intelligence
AMIA: American Medical Informatics Association
CHIMA: China Hospital Information Management Association
CHINC: China Hospital Information Network Annual Conference
CHITEC: China Health Information Technology Exchange Annual Conference
CMIAAS: China Medical Information Association Annual Symposium
CPMI: China Annual Proceeding of Medical Informatics
DALY: Disability-Adjusted Life Year
EHR: electronic health record
EMR: electronic medical record
GBD: Global Burden of Disease
HAC: Human, Animal, and Molecular/Cellular Biology
HIMSS: Healthcare Information and Management Systems Society
HIPAA: Health Insurance Portability and Accountability Act (HIPAA)
HIT: health information technology
HITECH: Health Information Technology for Economic and Clinical Health
ICD: International Classification of Diseases
ICT: information and communications technology
MeSH: Medical Subject Headings
MI: medical informatics
NIH: National Institutes of Health
WHO: World Health Organization
